# Longitudinal Listening Difficulties in Children Initially Presenting with Speech Sound Disorder: Two Case Reports

**DOI:** 10.3390/audiolres16050127

**Published:** 2026-08-28

**Authors:** Yoriko Fujimoto, Hirokazu Sakamoto, Tomoe Sekido

**Affiliations:** 1 Department of Rehabilitation, Osaka Metropolitan University Hospital, 1-5-7 Asahimachi, Abeno-ku, Osaka 545-8586, Japan; y-fujimoto@omu.ac.jp; 2 Department of Auditory and Language Information Pathophysiology, Graduate School of Medicine, Osaka Metropolitan University, 1-4-3 Asahimachi, Abeno-ku, Osaka 545-8585, Japan; osakamu_sekido_tomoe@omu.ac.jp

**Keywords:** listening difficulties, speech sound disorder, speech perception in noise, auditory processing, longitudinal follow-up

## Abstract

**Background and Clinical Significance:** Children may experience substantial listening difficulty (LiD) despite clinically normal pure-tone thresholds. The longitudinal course of LiD in children initially referred for speech sound disorder (SSD) is not well documented. **Case Presentation:** We retrospectively reviewed two individuals first evaluated during the preschool years for SSD and followed through adolescence or adulthood. Available preschool records showed air-conduction thresholds no poorer than 20 dB HL. Tympanometry showed type C1 in the right ear and type A in the left ear in Case 1 and bilateral type-A tympanograms in Case 2; bone-conduction thresholds and objective auditory tests were not documented in the reviewed records. Both cases later showed reduced word recognition in speech noise relative to their good performance in quiet, although the quiet and noise presentation levels were not identical in Case 2. A Japanese mishearing checklist yielded 6/60 in Case 1 and 24/60 by caregiver report and 45/60 by self-report in Case 2. Preschool and school-age assessments identified broader phonological, language, memory, or sequential-processing weaknesses. Serial Auditory Processing Test (APT) findings were heterogeneous. Original numerical source data were retrieved for all four serial APT assessments: Case 1 at ages 11 and 14 years and Case 2 at ages 16 and 20 years. All APT findings are interpreted descriptively, not as evidence of improvement or a diagnosis of auditory processing disorder (APD). Both cases received speech–language intervention. Case 1 later received school accommodations, whereas Case 2 continued to require compensatory strategies and adjustments in educational and workplace settings. **Conclusions:** These cases describe the co-occurrence of early speech-language difficulties and later clinically significant LiD but do not establish causality, predictive markers, or an APD diagnosis. Their value is descriptive: they show how functional listening needs and support requirements changed across development in two selected clinical cases.

## 1. Introduction

Listening difficulty (LiD) is an umbrella term for a reported difficulty in recognizing sounds or understanding speech, particularly under acoustically or linguistically demanding conditions [1]. Children with LiD may have clinically normal pure-tone thresholds yet struggle to understand speech in noise or reverberation, follow multistep oral instructions, process rapid or degraded speech, or respond consistently in everyday communication [1,2]. These difficulties can interfere with classroom participation, learning, and social communication. Importantly, listening is not a purely auditory act: successful speech understanding depends on interactions among peripheral and central auditory function, speech and language processing, attention, and memory [1,2,3].

Children with this clinical presentation have historically often been assessed within the framework of auditory processing disorder (APD). However, the diagnostic construct remains debated, and there is no universally accepted pediatric test battery or diagnostic criterion [1,4]. Large-sample and clinically referred studies have shown that performance on behavioral auditory processing tasks is associated with attention, working memory, nonverbal reasoning, language, and literacy [3,5]. Moreover, systematic review evidence indicates substantial overlap between children diagnosed with APD and children with other neurodevelopmental disorders, with few consistent features that clearly distinguish the groups [6]. Accordingly, we use LiD in this report as a symptom-based clinical descriptor and interpret auditory processing test results as evidence of vulnerability under specific task conditions, rather than as stand-alone proof of a discrete disorder.

This broader formulation has direct implications for assessment. A child’s reported problems should be examined using converging information from developmental and educational history, caregiver and self-report measures, peripheral hearing assessment, speech perception in quiet and noise, and selected auditory processing tasks [1,2,7]. Because the same observable complaint may arise from different combinations of auditory, phonological, linguistic, attentional, and memory-related factors, language and cognitive findings must be considered alongside audiological results [5,6,7]. Such multidimensional assessment is particularly important when the initial reason for referral concerns speech or language rather than hearing.

Speech sound disorder (SSD) is a heterogeneous condition involving difficulties in speech-sound perception, representation, planning, or production. Several features relevant to LiD—including unstable phonological representations, weak phonological awareness, reduced speech-perception accuracy, and limitations in auditory–verbal memory—may also occur in children with SSD. A recent systematic review identified recurring associations between SSD and auditory processing or phonological measures, while also emphasizing marked methodological heterogeneity and the risk of attributing language-related performance to an auditory deficit alone [8]. In addition, a cohort study of young school-aged children with a history of developmental phonological disorder found a higher proportion of caregiver-reported listening and processing difficulties than in typically developing peers [9]. These findings support careful clinical attention to possible overlap, but they do not establish that SSD causes later LiD or that early speech–language findings are predictive markers of an auditory disorder.

Most evidence concerning childhood LiD and APD has been cross-sectional or based on assessments conducted after school-age concerns were already established. In a prospective school-based questionnaire study of 743 students aged 6–18 years and their guardians at schools affiliated with Osaka Kyoiku University, student-reported LiD scores were higher in later school-year groups and diverged increasingly from guardian ratings, particularly in the later grades [10]. Because that analysis compared school-year groups rather than following the same children over time, it supports developmental differences in symptom reporting but does not establish within-child progression. Separate longitudinal work indicates that listening and associated cognitive difficulties can persist into adolescence in children with normal audiograms [11]. Nevertheless, detailed case-level descriptions that begin during the preschool years, when the presenting concern is SSD, and trace the subsequent emergence of LiD using serial functional, speech-in-noise, cognitive, and auditory processing findings remain limited. Such longitudinal descriptions cannot determine causality, but they can clarify when difficulties become clinically recognizable, how their expression changes as listening demands increase, and when environmental or compensatory support becomes necessary.

The aim of this case report is therefore to describe the longitudinal clinical course of two children who were initially followed in a speech–language clinic for SSD during the preschool years and later presented with clinically significant LiD. We focus on their early speech–language characteristics, the timing and context in which listening problems became apparent, the pattern and evolution of audiological and auditory processing findings, and the support required in school and adult life. We do not seek to establish a causal relationship between SSD and LiD, to validate diagnostic criteria for APD, or to propose early findings as predictors. Rather, these cases are presented to illustrate how longitudinal, multidimensional assessment may identify evolving listening needs that are not captured by pure-tone audiometry alone.

## 2. Materials and Methods

### 2.1. Design, Setting, and Ethics

This retrospective descriptive case report was based on longitudinal clinical records from a university hospital speech–language and audiology service. The report follows the CARE principles for transparent case reporting [12]. Both individuals were first evaluated during the preschool years because of unclear speech consistent with SSD and were subsequently reassessed when listening concerns became clinically relevant. Assessments were selected according to age and clinical need; consequently, the battery and interval were not uniform across time points. Chronological ages are stated when they were explicitly available in the record; otherwise, the developmental stage is reported to avoid false precision.

The study was approved by the Ethics Committee of Osaka Metropolitan University (approval No. 2021-215; latest amendment approved 9 April 2026) and was conducted in accordance with the Declaration of Helsinki. Written informed consent to participate in the study and for publication was obtained from the individuals and/or their guardians, as appropriate.

### 2.2. Peripheral Hearing and Speech Recognition in Quiet and Noise

Peripheral hearing was evaluated with age-appropriate air-conduction pure-tone audiometry. At the preschool assessment, play audiometry was used; bone-conduction thresholds were not documented. In Case 2, the initial record listed play-audiometry pure-tone averages of 13.8 dB HL for the right ear and 12.5 dB HL for the left ear. These averages were calculated using four-frequency method A: (threshold at 500 Hz + 2 × threshold at 1000 Hz + threshold at 2000 Hz)/4. Tympanometry was available at approximately 5 years of age. In Case 1, the right ear was classified as type C1 (peak pressure −111 daPa; peak compensated static admittance 0.62 mL; ear-canal volume 0.77 mL) and the left ear as type A (peak pressure −83 daPa; peak compensated static admittance 0.46 mL; ear-canal volume 0.82 mL). In Case 2, tympanograms were type A bilaterally (right: −30 daPa, 0.50 mL, 1.00 mL; left: −44 daPa, 0.60 mL, 0.90 mL, respectively). Acoustic reflexes, otoacoustic emissions, and auditory brainstem responses were not documented in the available records. A history of recurrent otitis media and hearing-aid use could not be retrospectively confirmed from the materials reviewed for this revision. Accordingly, the phrase clinically normal peripheral hearing in this report refers primarily to the available air-conduction thresholds and does not imply that a complete physiologic test battery was performed.

Speech recognition was measured in sound field using the Japanese 57-S monosyllabic word list. Clinical source graphs used the standard form labels “unaided” and “aided” to plot the quiet and speech-noise conditions, respectively; the clinical annotations identified the noise condition as speech and speech noise presented at a 0 dB signal-to-noise ratio (SNR). Percent-correct scores were calculated at the presentation levels recorded for each visit. In Case 1 at age 10 years, quiet and noise scores were both obtained at 40 dB, allowing for a same-level comparison. In Case 2 at age 16 years, the maximum quiet score and the 50 dB noise score were obtained at different presentation levels; therefore, they are reported descriptively and are not treated as a controlled 40-percentage-point within-level reduction. This sound-field 57-S assessment was separate from the multi-SNR APT speech-in-noise subtest described below.

### 2.3. Functional, Language, and Cognitive Assessment

Everyday listening was characterized using a Japanese mishearing checklist comprising 20 listening items rated from 0 to 3 (total score 0–60); higher scores indicate greater difficulty. A separate seven-item developmental-problem subscale is scored from 0 to 21. Scores in the range of 3–5 or higher on the listening scale have been proposed as a screening signal that warrants further evaluation, although the checklist is not diagnostic [13]. Caregiver report was obtained in both cases, and self-report was obtained when developmentally appropriate.

Speech–language and cognitive measures were selected from routine clinical tools and included the Illinois Test of Psycholinguistic Abilities (ITPA), the Wechsler Intelligence Scale for Children—Fourth Edition (WISC-IV), and the Kaufman Assessment Battery for Children—Second Edition (KABC-II). These measures were used to describe phonological, auditory–verbal memory, language, sequential-processing, and broader cognitive characteristics. They were not used to infer that one domain caused the listening symptoms.

### 2.4. Auditory Processing Test and Interpretation

Selected subtests from the Japanese Auditory Processing Test (APT) battery were administered using the clinical protocol described in the Japanese LiD/APD literature [13,14]. The battery can include monaural speech tasks, dichotic word and sentence listening, fast-speech perception at normal, 1.5-times, and 2-times rates, word recognition in speech noise across SNRs of +10, +5, 0, −5, −10, and −15 dB, gap detection, auditory attention, alternating binaural listening, and competing-speech tasks. Only the subtests clinically indicated and developmentally feasible at each visit were administered; therefore, serial comparisons were restricted to conditions available at both time points.

APT scores were interpreted descriptively and in conjunction with the functional, language, cognitive, and audiological data. Original numerical source data were retrieved during revision for both Case 1 APT assessments (ages 11 and 14 years) and both Case 2 assessments (ages 16 and 20 years). Because validated age-specific pediatric normative values were unavailable for all conditions, pediatric scores were interpreted relative to the clinical reference ranges used at the time, and no APT result was used as a stand-alone diagnostic cutoff for APD. Published Japanese normative data for the Obuchi APT were derived initially from 20 normal-hearing adults aged 20–37 years [14], and subsequent work has shown the effects of age and tested side effects in a larger adult sample [15]. These adult reference values were not applied to diagnose the children; they were used only as descriptive context for adult follow-up. The −10 and −15 dB SNR conditions were considered susceptible to floor effects because low scores occur even in normal-hearing adults [14].

### 2.5. Analysis

Because this was a two-case descriptive report, no inferential statistical analysis was performed. We examined within-person differences across assessments, concordance and discordance among objective and subjective measures, and the functional circumstances in which support was required.

### 2.6. Use of Generative Artificial Intelligence

During preparation of this manuscript, the authors used ChatGPT 5.6 (OpenAI; accessed July–August 2026) to assist with English-language editing, drafting of portions of the text, and preparation of publication-ready figure layouts from author-supplied clinical values and source graphs. It was not used to generate or analyze clinical data or to make clinical decisions. All output was critically reviewed, verified against the clinical records, source graphs, and cited literature, and revised by the authors, who take full responsibility for the content of the publication.

## 3. Case Presentations

**Figure 1:**
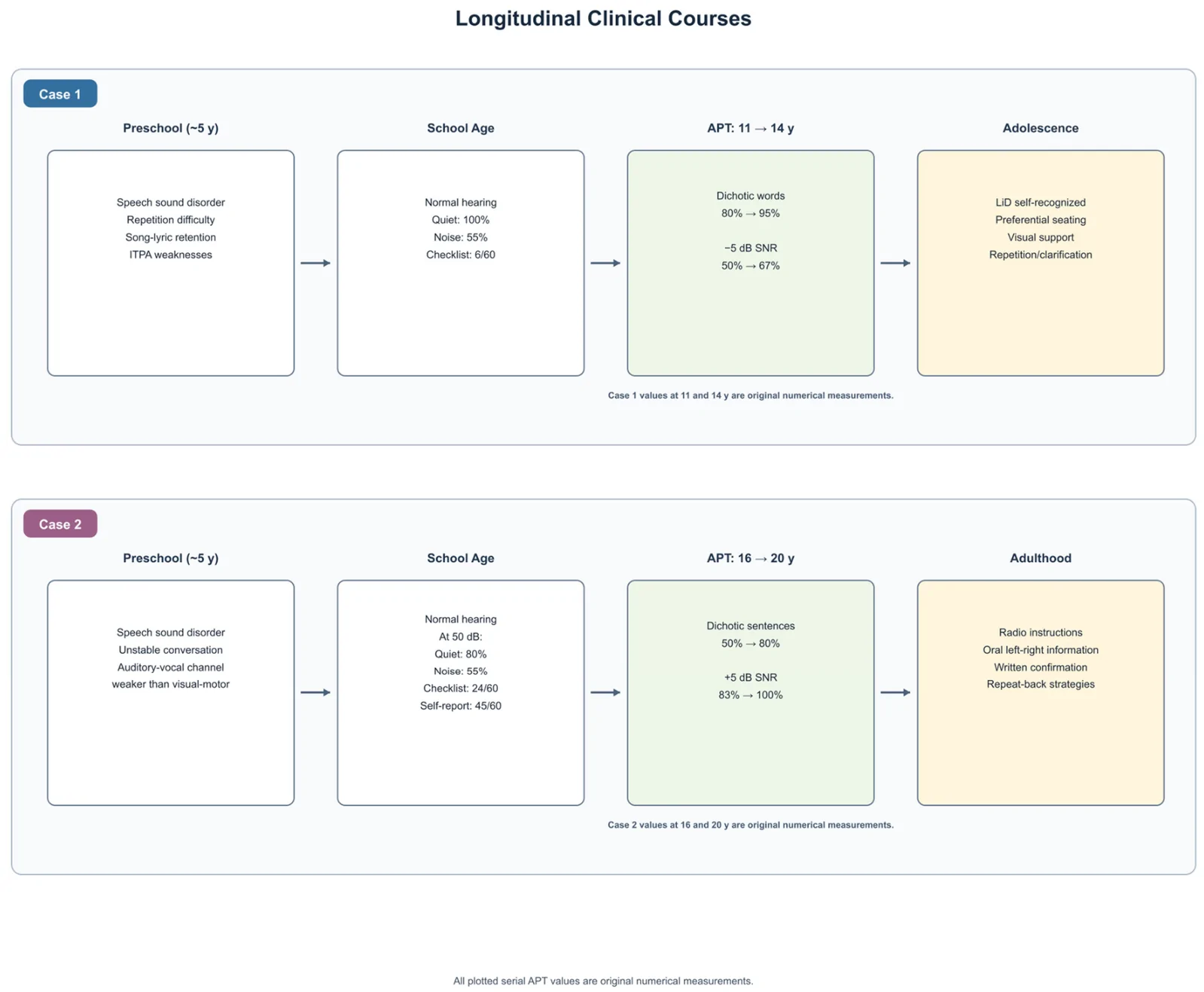
Longitudinal clinical courses of the two cases. Case 1: from preschool speech–language presentation through school-age multidimensional assessment, serial APT at ages 11 and 14 years, and adolescent support. Case 2: from preschool speech–language presentation through school-age LiD assessment, serial APT at ages 16 and 20 years, and adult functional needs. All displayed serial APT percentages are original numerical measurements. APT, Auditory Processing Test; LiD, listening difficulty; PTA, pure-tone average; SNR, signal-to-noise ratio; WMI, Working Memory Index.

The longitudinal clinical courses of the two cases are summarized in Figure 1.

### 3.1. Case 1

Case 1 was male and first attended the speech–language clinic at 5 years 4 months (19 September 2013) because of unclear speech. The clinical diagnostic labels entered at the initial visit were articulation disorder and language developmental delay. Recorded speech-sound errors included /s/ produced as /ʃ/, /r/ produced with an immature realization or as /d/, and /ga/ produced as /ka/ despite other /g/ productions being correct. The record transcribed one /dz/ error as “d3”; the intended phonetic symbol could not be retrospectively confirmed. Word-level speech was frequently described as unclear. The available examples suggested variable phonological-form errors rather than a single invariant substitution: /megane/ was produced as /megame/ at one visit and /sakana/ as /kasana/ at another, and a referral attachment stated that isolated sounds could be repeated but errors increased when different sounds were sequenced and as syllable length increased. The record also referred to immature phonological development. Developmental history recorded no concern at the 18-month or 3.5-year health examinations, several first words by approximately 1 year 6 months, subsequent vocabulary growth, and difficulty producing longer sentences until early preschool. He could hum melodies but had difficulty reproducing song lyrics. Gross-motor development and peer relationships were described as unremarkable, with mild fine-motor clumsiness. However, a standardized SSD subtype, formal severity or intelligibility rating, and a differential evaluation for developmental language disorder or childhood apraxia of speech were not documented and could not be retrospectively confirmed. The initial oral and pharyngeal examination was unremarkable; soft-palate elevation and tongue movements were documented as normal.

Several first words were reported by approximately 1 year 6 months, although the exact age of the first word was not documented; the 18-month and 3.5-year health checks had been passed. At approximately 5 years, two-word utterances were present; a contemporaneous evaluation estimated receptive language at 4 years 2 months and expressive language below the 3 years 1 month item level, with performance considered to be in the 2-year range. At 5 years 4 months, the Picture Vocabulary Test—Revised yielded a vocabulary age of 5 years 0 months and a standard score of 9. At 5 years 6 months, everyday questions and riddles were at the 5-year level, whereas category concepts, word definitions, and verbal analogy were lower. Partial ITPA testing at 5 years 8 months showed contrasting age-equivalent scores: word comprehension 6 years 3 months, picture comprehension 6 years 2 months, verbal analogy 3 years 11 months, picture analogy 5 years 7 months, number memory 2 years 10 months, shape memory 8 years 0 months, and visual closure 5 years 1 month. These results documented an uneven language-processing profile but were not used retrospectively to assign DLD, CAS, LiD, or APD.

Speech–language therapy began at 5 years 4 months and ended at 14 years 11 months. Visits were most frequent at approximately monthly intervals, with some periods of two-month spacing or observation over approximately six months. Documented intervention included phonological segmentation and identification of initial sounds. At 5 years 6 months, home guidance emphasized modeling complete two- to three-word utterances, particularly avoiding omission of verbs. The language conference record dated 5 April 2023 documented termination because language performance was considered good. The available retrospective record did not provide sufficient data on treatment dose or repeated measures of speech severity to separate intervention effects from maturation or other developmental change.

At 5 years, play audiometry showed air-conduction thresholds of approximately 5–20 dB HL across the tested frequencies. Tympanometry showed right-type C1 and left-type A, as described above. During school age, air-conduction thresholds remained within the clinically normal range; the recorded pure-tone averages calculated using four-frequency method A were 13.8 dB HL in the right ear and 8.8 dB HL in the left ear. At age 10, sound-field word recognition was 100% in quiet and 55% in 0 dB SNR speech noise, both at 40 dB. At 50 dB, subsequent quiet/noise scores were 96%/50% at age 13, 94%/54% at age 15, 94%/62% at age 16, and 92%/72% at age 17. These serial values document variable performance in noise and do not establish progressive improvement. The 2017 clinical summary recorded a caregiver-completed mishearing checklist score of 6/60 and a developmental-problem subscale score of 0/21. It also recorded a WISC-IV Full-Scale IQ of 76, with Verbal Comprehension Index 72, Perceptual Reasoning Index 76, Working Memory Index 82, and Processing Speed Index 94. The same summary listed an earlier KABC-II Mental Processing Index of 69 (Sequential 65, Simultaneous 71, Planning 68, Learning 97) and an Achievement Scale score of 69 (Vocabulary 77, Reading 67, Writing 64, Mathematics 71). On the later KABC-II administered at 11 years 2 months, the Mental Processing Index was 76 (Sequential 72, Simultaneous 77, Planning 73, Learning 102), and the Achievement Scale score was 72 (Vocabulary 90, Reading 67, Writing 71, Mathematics 70). Thus, the listening profile occurred in the context of broader language and cognitive variability rather than as an isolated auditory finding.

**Figure 2:**
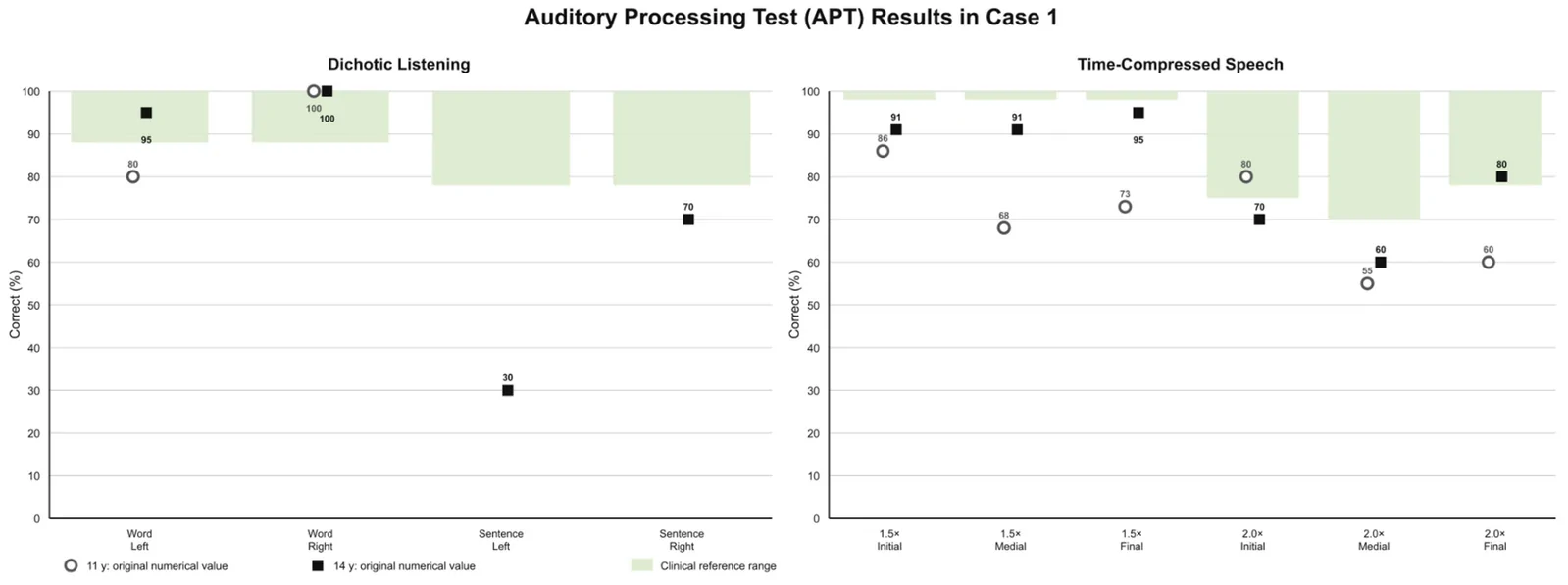
Auditory Processing Test results in Case 1 at ages 11 and 14 years. Dichotic word and sentence listening are shown in the left panel, and time-compressed speech results at 1.5-times and 2-times rates are shown in the right panel. Open circles indicate age 11, filled squares indicate age 14, and pale green shading reproduces the reference range shown on the original clinical APT record; it was not treated as an age-specific pediatric diagnostic cutoff. APT, Auditory Processing Test. Open circles show original numerical measurements from the retrieved age-11 summary sheet; dichotic-sentence values were not entered on that sheet. Filled squares show original numerical measurements from the retrieved age-14 summary sheet. The serial differences are not evidence of true longitudinal change.

**Figure 3:**
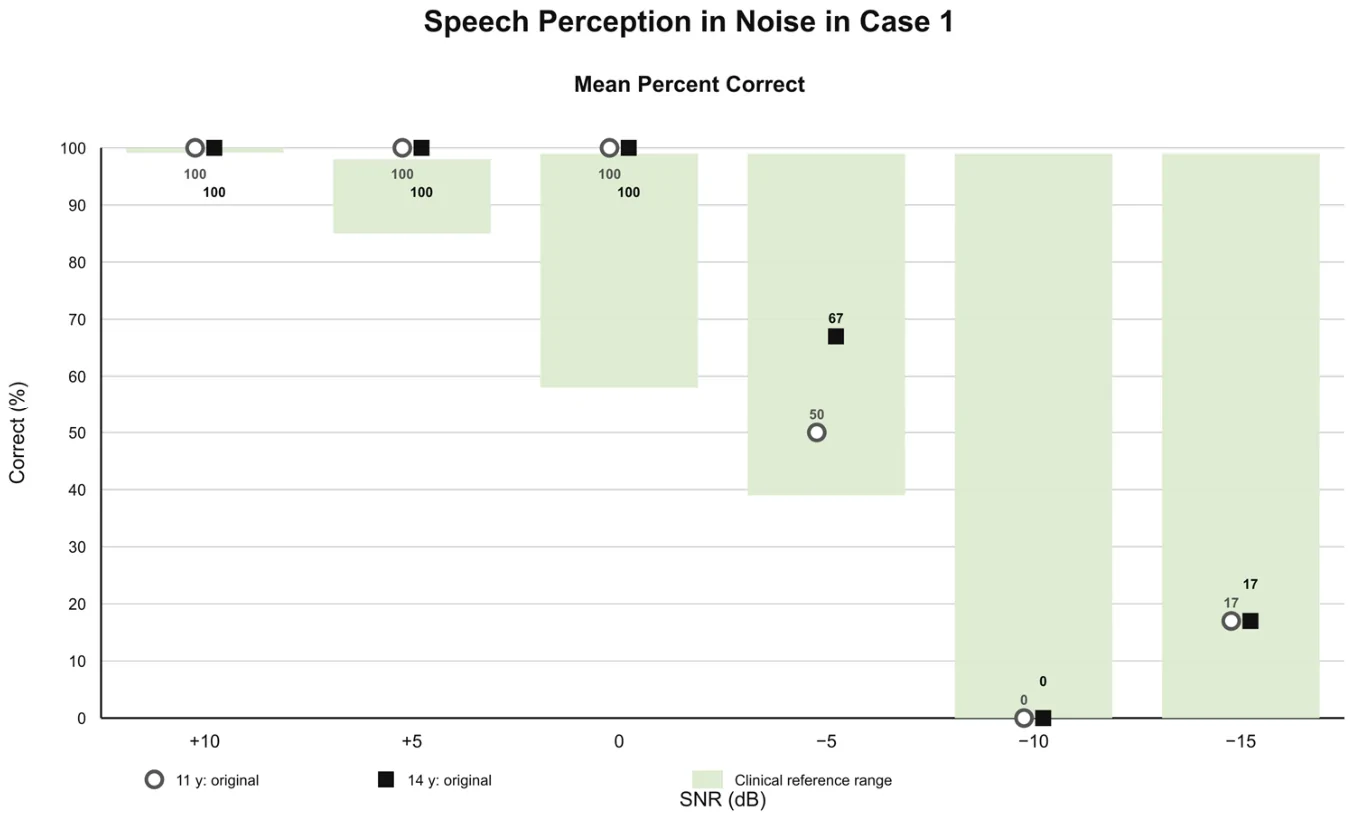
Speech perception in noise in Case 1 at ages 11 and 14 years. Percent-correct scores are plotted across SNRs from +10 to −15 dB. Open circles indicate age 11, filled squares indicate age 14, and pale green shading reproduces the reference range shown on the original clinical APT record; it was not treated as an age-specific pediatric diagnostic cutoff. SNR, signal-to-noise ratio. Open circles show original numerical measurements from the retrieved age-11 summary sheet. Filled squares show original numerical measurements from the retrieved age-14 summary sheet. The serial differences are not evidence of true longitudinal change.

APT was performed at 11 years and repeated at 14 years (Figure 2). Original numerical summary sheets for both assessments were retrieved during revision. At age 11, dichotic-word scores were 80% for the left ear and 100% for the right ear; dichotic-sentence values were not entered. At 1.5-times speed, initial-, medial-, and final-word scores were 86%, 68%, and 73%, respectively; at 2-times speed, the corresponding scores were 80%, 55%, and 60%. Speech-in-noise scores at +10, +5, 0, −5, −10, and −15 dB SNR were 100%, 100%, 100%, 50%, 0%, and 17%, respectively (Figure 3). Alternating binaural listening was 90%, and the gap-detection threshold was 34 ms. At age 14 (30 March 2023), dichotic-word scores were 95% for the left ear and 100% for the right ear, and dichotic-sentence scores were 30% and 70%, respectively. At 1.5-times speed, initial-, medial-, and final-word scores were 91%, 91%, and 95%; at 2-times speed, they were 70%, 60%, and 80%. Speech-in-noise scores at +10, +5, 0, −5, −10, and −15 dB SNR were 100%, 100%, 100%, 67%, 0%, and 17%, respectively (Figure 3). Multiple-speech listening was 95%, the gap-detection threshold was 2 ms, and the auditory attention task was 100% correct (20/20; mean correct-response time recorded as 504, with no unit printed on the source sheet). The alternating binaural-listening field was not completed. These between-visit differences were not interpreted as genuine improvement because maturation, familiarity, practice effects, and measurement variability could not be separated. Performance was near ceiling level at favorable or neutral SNRs, whereas the −10 and −15 dB conditions were at floor or near floor level and were not interpreted diagnostically. Dichotic sentence and fast-speech conditions remained vulnerable in the serial records. The APT findings therefore describe task conditions under which performance was less robust; they do not demonstrate deterioration, recovery, or APD.

The individual’s own awareness of listening difficulty became clearer during junior high school, when classroom speech became faster, oral information increased, and listening environments became more complex. School accommodations included preferential seating, reduction in avoidable background noise, visual supplementation of oral instructions, repetition of key information, and explicit permission to request clarification.

### 3.2. Case 2

Case 2 was female and first attended the speech–language clinic at 5 years 6 months on 21 August 2008 because of unclear speech. The initial note contained the clinical terms language delay/language disorder and articulation disorder, but did not document a standardized diagnostic formulation. The 18-month health examination was reported as unremarkable; the 3-year examination was not attended. Independent walking occurred at 11 months, and no gross- or fine-motor concern was recorded. Several first words were present by approximately 1 year 6 months, and vocabulary subsequently increased, although her speech remained difficult to understand. At the preschool evaluation, the S-S method showed word-order comprehension but not established particle comprehension; expressive speech was at sentence level. The Picture Vocabulary Test—Revised yielded a vocabulary age of 4 years 9 months. The ITPA yielded a language-learning age of 5 years 8 months and a mean standard score of 36.3, with the auditory–vocal channel weaker than the visual–motor channel. Recorded speech-sound errors involved sibilant, affricate, and palatalized consonants. Examples transcribed in the source record included /s/ produced as /t/ or /tʃ/, /ts/ as /tʃ/, /ʃ/ as /tj/ or /tʃ/, and /kj/ as /tj/, /tʃ/, or /t/. The record used the notation “d3” for some /dz/ and /gj/ errors; the intended phonetic symbol could not be retrospectively confirmed. For /ra/ and /re/, an atypical circular tongue movement was noted. Additional word-level phonological errors were documented. Because alternative outputs were listed for some targets, variability was suggested, but consistency was not formally assessed. Nasality was absent, and no abnormality was documented in velopharyngeal closure or tongue movement. The caregiver reported difficulty biting through meat, but no feeding or swallowing disorder was documented. Formal standardized SSD subtype and severity ratings and a formal differential assessment for developmental language disorder or childhood apraxia of speech were not documented and could not be retrospectively confirmed. Kana reading was emerging by late preschool and was largely established at the initial visit; peer relationships were described as unremarkable.

The historical clinical-course record documented speech–language intervention from the final preschool year through junior high school. Because the family traveled from a distance, sessions during the documented preschool-to-school-age phases were generally scheduled once or twice per month. Intervention targets included production of affected consonants, auditory discrimination, phonological segmentation and sound extraction, word repetition, and later use of written or reading cues to support accurate production. At 7 years 7–8 months, clinical training records documented successful two-choice discrimination of /ki/ versus /tʃi/, /kja/ versus /tʃa/, and /kju/ versus /tʃu/, whereas responses became ambiguous when six similar alternatives were presented. The same records linked unstable phonological forms with reading and writing errors and described reduced auditory retention. At 10 years 8 months, she could discriminate the charted “dz/d3” and /s/-/ʃ/ contrasts and practiced elicitation of /dza/ and /dzu/ at word level; the source notation “d3” could not be retrospectively resolved to a specific phonetic symbol. Preschool ITPA results showed number memory below shape memory (standard scores 30 and 41, respectively); a later ITPA profile again showed lower number memory than shape memory (27 and 41). Repetition of words of three or more syllables remained unclear. At 13 years 10 months, PVT-R yielded a standard score of 4, and the record documented phonologically related dictation errors and described weak working-memory capacity. School-age records also described difficulty keeping up with dictation, hearing the teacher, learning or recalling spoken word forms, and writing words accurately from speech. These findings document broader phonological and auditory–verbal vulnerabilities but do not establish DLD, CAS, LiD, or APD retrospectively. By the end of fifth grade, all target sounds could be produced at word level and in carefully monitored sentences, although mislearning of spoken word forms and writing errors persisted. Therapy was temporarily discontinued for circumstances not specified in the source record, and reassessment occurred in the first junior-high-school year after approximately one year. Exact session duration, repeated standardized speech-severity measures, and the relative contributions of intervention and maturation were not documented in the source available for this revision.

At 5 years 6 months, the play-audiometry pure-tone averages calculated using four-frequency method A were 13.8 dB HL for the right ear and 12.5 dB HL for the left ear. Tympanograms were type A bilaterally. At age 16, the pure-tone averages were 6.7 dB HL right and 8.3 dB HL left by the three-frequency method, 6.3 and 7.5 dB HL by four-frequency method A, and 7.5 and 8.3 dB HL by the six-frequency method. The previously recorded school-age pure-tone averages calculated using four-frequency method A were 11.3 dB HL right and 6.3 dB HL left. Sound-field results were variable across visits. At age 15, the best quiet score was 100% at 40 dB and the noise score was 10% at 40 dB. At age 16, the maximum quiet score was 95% at 40 dB; at 50 dB, the quiet and noise scores were 80% and 55%, respectively. At age 20, quiet and noise scores at 50 dB were 96% and 28%, respectively. These results document persistent susceptibility to speech noise but substantial visit-to-visit variability; they do not support a monotonic trajectory. The 2017 clinical summary recorded mishearing checklist scores of 24/60 by caregiver report and 45/60 by self-report. Cognitive assessment showed a WISC-IV Full-Scale IQ of 90, with Verbal Comprehension Index 93, Perceptual Reasoning Index 104, Working Memory Index 76, and Processing Speed Index 88. The same 2017 clinical summary reported a KABC-II Mental Processing Index of 80 (Sequential 76, Simultaneous 107, Planning 84, Learning 69) and an Achievement Scale score of 73 (Vocabulary 72, Reading 69, Writing 79, Mathematics 90); the exact KABC-II test date was not shown in that summary. A separately retrieved clinical note dated 17 March 2015 (age 12 years 1 month) recorded the following subtest scaled scores (the Japanese labels are translated descriptively): word learning 1, digit span 7, picture integration 12, word order 7, hand movements 8, expressive vocabulary 6, and numerical reasoning 9. Whether the summary profile and the dated partial record represented the same assessment episode could not be retrospectively confirmed. At age 20, WMS-R Logical Memory I yielded a raw score of 22 (22nd percentile), with errors involving names, places, and amounts. These data require a multidimensional interpretation that includes auditory–verbal memory and sequential processing.

**Figure 4:**
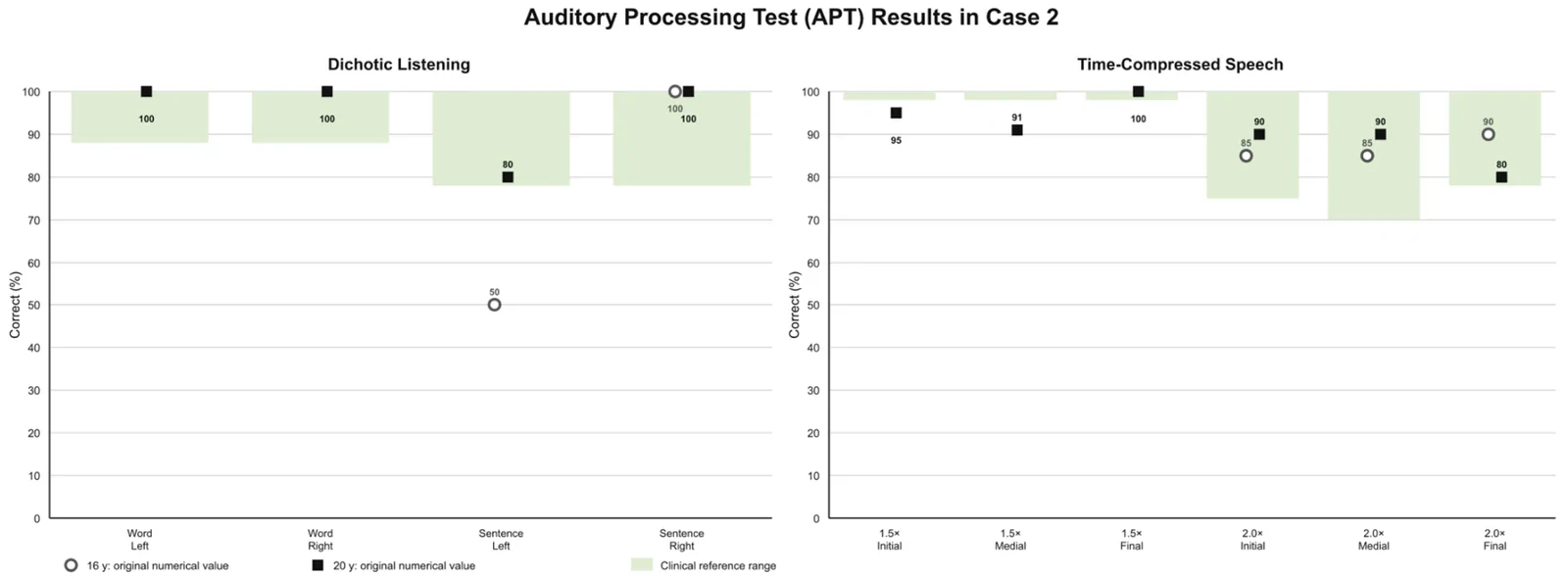
Auditory Processing Test results in Case 2 at ages 16 and 20 years. Open circles show original numerical measurements from the age-16 source record; dichotic-word and 1.5-times-speed fields were not completed. Filled squares show original numerical measurements from the age-20 source record. The serial differences are not evidence of true longitudinal change.

**Figure 5:**
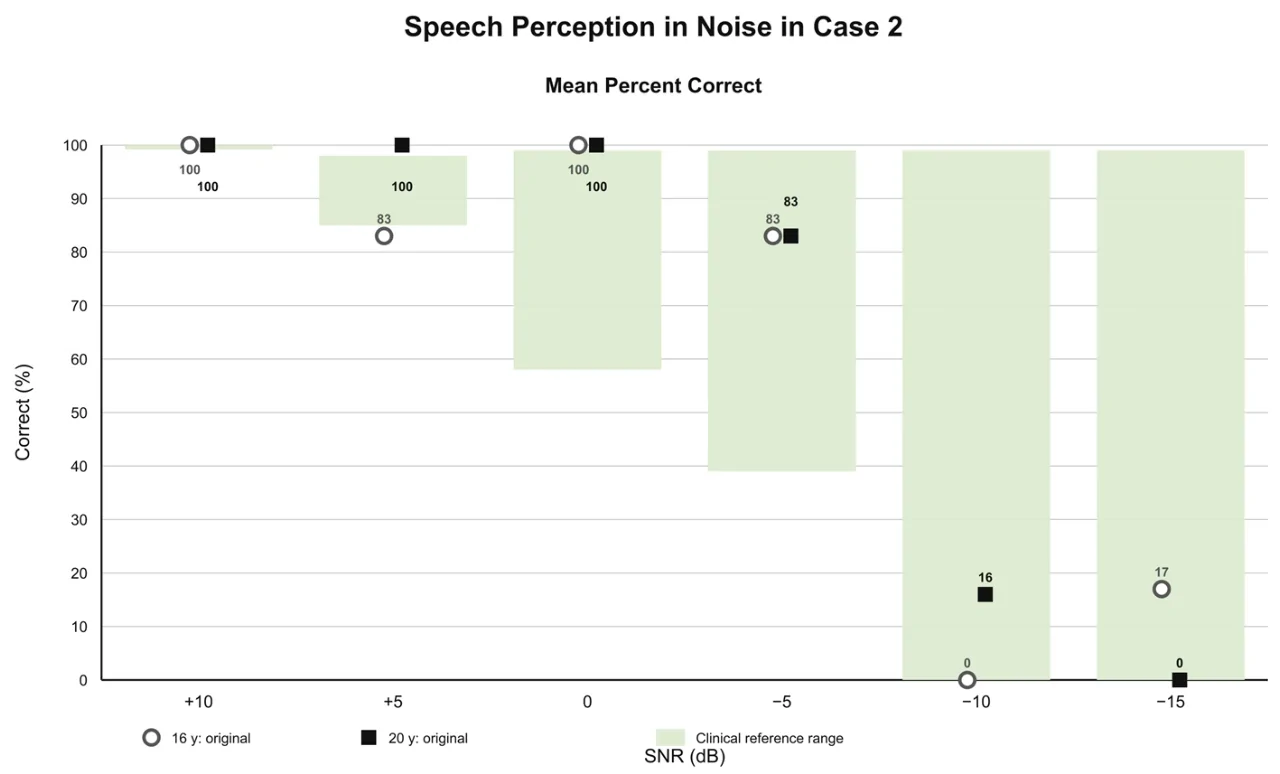
Speech perception in noise in Case 2 at ages 16 and 20 years. Open circles show original numerical measurements from the age-16 source record, and filled squares show original numerical measurements from the age-20 source record. The serial differences are not evidence of true longitudinal change.

APT was performed at age 16 (31 July 2019) and repeated at age 20 (26 September 2023) (Figure 4). Original numerical source data were retrieved for both assessments. At age 16, dichotic-sentence scores were 50% for the left ear and 100% for the right ear; dichotic-word fields were not completed. At 2-times speed, initial-, medial-, and final-word scores were 85%, 85%, and 90%, respectively; 1.5-times-speed fields were not completed. Speech-in-noise scores at +10, +5, 0, −5, −10, and −15 dB SNR were 100%, 83%, 100%, 83%, 0%, and 17%, respectively (Figure 5). Multiple-speech listening was 65%, the gap-detection threshold was 2 ms, and the source record listed a 100% correct rate for the auditory-attention task (20 correct responses) together with two errors. At age 20, dichotic-word scores were 100% bilaterally, and dichotic-sentence scores were 80% for the left ear and 100% for the right ear. At 1.5-times speed, initial-, medial-, and final-word scores were 95%, 91%, and 100%; at 2-times speed, they were 90%, 90%, and 80%. Speech-in-noise scores at +10, +5, 0, −5, −10, and −15 dB SNR were 100%, 100%, 100%, 83%, 16%, and 0%, respectively (Figure 5). Multiple-speech listening was 90%, the gap-detection threshold was 2 ms, and the auditory-attention form listed an 85% correct rate (17 correct responses) and 0 errors. Alternating-binaural-listening fields were not completed at either assessment. Between-visit differences were not interpreted as genuine improvement because maturation, familiarity, practice effects, and measurement variability could not be separated. At −10 and −15 dB SNR, performance remained at floor level or near floor level, limiting interpretation. The APT findings are therefore presented descriptively as task-specific vulnerabilities and do not demonstrate longitudinal improvement, deterioration, or APD.

Listening-related educational and occupational needs persisted beyond compulsory schooling. During later school years, the record described difficulty hearing the teacher in noise, following spoken English, and keeping pace with dictation; the family consulted an education center and sought environmental adjustment. Before high-school entry, a parent explained her listening characteristics during school consultation. At 13 years 10 months, the clinical record noted that she found speech difficult to understand in noisy settings; school measures included seating within the first three rows and away from particularly noisy classmates. At 15 years 10 months, a parking-job record noted that she received instructions through an earpiece and could accurately hear telephone numbers; later functional history nevertheless included difficulty understanding radio communications. At an animal-care vocational school, she sometimes missed spoken information or could not identify key points and used two notebooks to record and later reorganize practicum information. During driver-license training, she reported missed or misheard instructions and confusion between orally presented left and right directions. At age 20, she worked five days per week in an animal-related part-time job and reported difficulty retaining telephone messages and speech presented in instructional videos; she used screenshots and written notes as compensatory strategies. These examples document real-world participation needs; the available record did not provide standardized measures of the effectiveness of individual accommodations.

### 3.3. Cross-Case Summary

Both cases had clinically normal air-conduction thresholds and good word recognition in quiet but showed reduced performance in speech noise. A same-level quiet/noise comparison was available for Case 1 at age 10 (100% versus 55% at 40 dB). In Case 2, quiet and noise scores varied by presentation level and visit; at age 16 the same-level 50 dB scores were 80% and 55%, respectively. Both cases also had early speech–language or broader neurodevelopmental findings that could contribute to test performance. The shared feature was therefore a functional LiD phenotype in two selected clinical cases, not a uniform APD test pattern or evidence that SSD predicted later LiD. Key longitudinal findings are summarized in Table 1.

### 3.4. Patient Perspective

A standalone patient-perspective statement was not collected because this report was reconstructed retrospectively from clinical records. However, the individuals’ own accounts are represented in the descriptions of increasing self-awareness, the self-report checklist, and the school and workplace situations in which listening difficulty affected participation.

## 4. Discussion

This report provides a descriptive account of two selected individuals who first presented with SSD during the preschool years and later experienced clinically significant LiD. Its contribution is not a new diagnostic criterion, a causal model, or a universal follow-up recommendation. Rather, it adds case-level detail concerning the changing clinical presentation, serial speech-in-noise performance, speech–language intervention, APT limitations, and functional support needs across childhood, adolescence, and adulthood. The two courses were heterogeneous and do not demonstrate progressive auditory decline or a common developmental pathway.

The early SSD and later LiD findings should be interpreted as co-occurring clinical features rather than as a proven developmental sequence. SSD is heterogeneous, and speech perception, phonological representation, auditory–verbal memory, motor planning, and language may contribute in different proportions [8,16,17]. Recent work has reported greater caregiver-rated listening and processing difficulty in some children with developmental phonological disorder [9], but available evidence does not establish sensitivity, specificity, or predictive value for later LiD. In the present cases, weaknesses involving repetition, phonological processing, auditory–verbal memory, working memory, or sequential processing were documented before or alongside later listening complaints. These case-level observations support further prospective study but do not establish an early marker, causal pathway, or indication to screen all children with SSD for LiD.

The findings also support maintaining a clear distinction between the symptom of LiD and the diagnosis of APD. Behavioral auditory tasks require the child to attend, understand instructions, retain verbal material, select a response, and often use phonological or lexical knowledge [1,3,5,6]. Case 1 had a Full-Scale IQ of 76 with variable index scores, and both cases showed working-memory or sequential-processing weaknesses. These factors may have contributed to APT performance and cannot be removed analytically in a two-case retrospective report. Accordingly, we did not diagnose APD from a count of subtests below a reference value. We used LiD to describe the everyday complaint and interpreted APT results as a map of conditions under which performance was more or less robust.

Speech-in-noise testing added information not captured by the audiogram or quiet word recognition, but the longitudinal data require careful, condition-specific interpretation. In Case 1, the age-10 quiet/noise comparison was obtained at the same 40 dB presentation level. In Case 2, the frequently reported values of 95% in quiet and 55% in noise came from different presentation levels; the same-level 50 dB scores at age 16 were 80% and 55%. Later observations showed variable noise scores rather than a monotonic pattern. Very adverse APT SNR conditions also showed floor effects. Accordingly, neither a single quiet–noise difference nor change across visits should be equated with progression or resolution of LiD.

Subjective recognition and functional demands changed across development. In Case 1, the listening complaint became salient during adolescence rather than at the preschool presentation. In Case 2, self-report exceeded caregiver report by 21 points, and practical difficulty remained relevant in adulthood despite between-visit differences in serial APT measurements. In a prospective survey of 743 student–guardian pairs, student-reported LiD scores were higher in later school-year groups, whereas guardians more often reported no difficulty; the discrepancy was greatest in the later grades [10]. This school-year association is consistent with the possibility that increasing listening demands and developing self-awareness influence when LiD is reported, although the survey was not a within-child longitudinal study. The divergence between test performance, observer report, and self-report also supports a multidimensional interpretation in which real-world listening reflects both individual characteristics and environmental demands [1,2,7]. Separate longitudinal evidence indicates that childhood listening and associated cognitive difficulties may persist into adolescence [11]. In clinical practice, reassessment can therefore be considered when functional concerns emerge or listening demands materially change; these two cases do not establish a fixed reassessment schedule.

Management in these cases was based on the functional profile. Preferential seating, reduction in competing noise, visual supplementation, shorter segmented instructions, repetition of key information, and permission to request clarification were low-risk accommodations aligned with clinical guidance [4]. Remote-microphone technology can improve perceived classroom listening for some children diagnosed with APD [18], but it was not evaluated systematically in these two cases and should not be inferred as a study outcome. In adulthood, written confirmation, repeat-back, and explicit checking of laterality helped reduce the consequences of missed or reversed instructions. The target of intervention was participation and accuracy, not normalization of a diagnostic label or a single score.

Several limitations constrain interpretation. First, the report includes only two individuals referred to a specialized speech–language clinic, creating substantial selection bias and precluding generalization to children with SSD or LiD as a group. Second, the retrospective design prevented standardized assessment intervals, and the battery varied with age and clinical need. Speech–language therapy was provided for both cases, but treatment dose and repeated speech-severity data were insufficient to distinguish treatment effects from maturation. Third, a standardized SSD subtype and severity rating and formal retrospective differentiation from DLD or CAS were unavailable. Fourth, the scope of peripheral hearing documentation was incomplete: bone conduction, acoustic reflexes, otoacoustic emissions, and auditory brainstem responses were not available. Fifth, validated pediatric diagnostic cutoffs were unavailable for several APT conditions. Original numerical source data were retrieved for all four serial APT assessments, but this does not remove the limitations of nonstandardized intervals, incomplete fields at some visits, and age-dependent task performance. APT performance is also influenced by language, attention, memory, and cognition, and repeated testing may introduce practice effects. Finally, there was no control group or design capable of determining whether early SSD and later LiD were causally related, reflected a shared vulnerability, or occurred independently. Prospective studies using standardized intervals, age-appropriate norms, functional outcomes, and comparison groups are required.

## 5. Conclusions

These two cases describe clinically important LiD that became apparent after preschool referral for SSD, in the presence of clinically normal air-conduction thresholds and broader speech–language or cognitive vulnerabilities. The observations do not establish that SSD predicts LiD, that serial APT differences represent clinical improvement, or that either individual met diagnostic criteria for APD. Follow-up decisions should therefore be individualized and triggered by persistent functional concerns—such as difficulty following spoken information, understanding speech in noise, or coping with increased educational or occupational listening demands—rather than by SSD alone or by a fixed schedule. Prospective studies are required before specific screening criteria or reassessment intervals can be recommended.

## Figures and Tables

**Table 1:** Longitudinal clinical and assessment summary.

Domain	Case 1	Case 2
Initial presentation	Male; articulation disorder and language developmental delay at 5 years 4 months; variable phonological-form errors; standardized SSD subtype and severity not documented	Female; language delay/language disorder and articulation disorder noted at 5 years 6 months; variable sibilant, affricate, and palatalized-consonant errors; standardized SSD subtype and severity not documented
Pure-tone hearing	Age 5 air conduction approximately 5–20 dB HL; tympanometry right C1/left A; later PTA 13.8 dB HL right, 8.8 dB HL left (four-frequency method A); bone conduction and physiologic tests not documented	Age 5 years 6 months play-audiometry PTA 13.8 dB HL right/12.5 dB HL left (four-frequency method A); tympanometry A/A; age 16 PTA 6.3/7.5 dB HL (four-frequency method A; 3-frequency 6.7/8.3 dB HL); bone conduction and physiologic tests not documented
Sound-field words	Age 10: quiet 100%, 0 dB SNR noise 55% at the same 40 dB level; age 13–17 noise scores 50–72% at 50 dB	Age 16: quiet maximum 95% at 40 dB; at 50 dB quiet 80% and 0 dB SNR noise 55%; age 20 quiet/noise 96%/28% at 50 dB
Mishearing checklist	Caregiver 6/60; developmental subscale 0/21 (2017 clinical summary)	Caregiver 24/60; self-report 45/60 (2017 clinical summary)
Language/cognition	Uneven preschool language profile; ITPA verbal analogy and number memory low; WISC-IV FSIQ 76, VCI 72, PRI 76, WMI 82, PSI 94; earlier 2017 KABC-II summary: MPI 69 (Sequential 65, Simultaneous 71, Planning 68, Learning 97), Achievement 69 (Vocabulary 77, Reading 67, Writing 64, Mathematics 71); later KABC-II at 11 years 2 months: MPI 76 (Sequential 72, Simultaneous 77, Planning 73, Learning 102), Achievement 72 (Vocabulary 90, Reading 67, Writing 71, Mathematics 70)	ITPA language-learning age 5 years 8 months, mean SS 36.3, auditory–vocal weaker than visual–motor; WISC-IV FSIQ 90, VCI 93, PRI 104, WMI 76, PSI 88; 2017 KABC-II summary: MPI 80 (Sequential 76, Simultaneous 107, Planning 84, Learning 69), Achievement 73 (Vocabulary 72, Reading 69, Writing 79, Mathematics 90); dated age-12 partial note listed subtest scaled scores 1, 7, 12, 7, 8, 6, and 9; relationship between records unconfirmed
Serial APT	Original numerical APT values available at ages 11 and 14; serial differences not interpreted as improvement	Original numerical APT values available at ages 16 and 20; selected dichotic, fast-speech, and speech-in-noise conditions remained vulnerable; low-SNR floor effects; serial differences not interpreted as improvement
Functional course	Speech–language therapy from 5 years 4 months to 14 years 11 months; later self-awareness and school accommodations	Speech–language intervention from late preschool through junior high, generally once or twice monthly during documented phases; educational consultation; radio-communication, vocational-school, and orally presented left-right difficulties in adulthood

Note: Case 1 age-11 and age-14 APT values and Case 2 age-16 and age-20 APT values are original numerical measurements. APT, Auditory Processing Test; FSIQ, Full-Scale IQ; ITPA, Illinois Test of Psycholinguistic Abilities; KABC-II, Kaufman Assessment Battery for Children—Second Edition; MPI, Mental Processing Index; PTA, pure-tone average; SNR, signal-to-noise ratio; SSD, speech sound disorder; WMI, Working Memory Index.

## Data Availability

The clinical data supporting this report are not publicly available because they contain information that could compromise participant privacy. De-identified information may be available from the corresponding author upon reasonable request and subject to institutional and ethical restrictions.
